# Treatment estimands in clinical trials of patients hospitalised for COVID-19: ensuring trials ask the right questions

**DOI:** 10.1186/s12916-020-01737-0

**Published:** 2020-09-09

**Authors:** Brennan C. Kahan, Tim P. Morris, Ian R. White, Conor D. Tweed, Suzie Cro, Darren Dahly, Tra My Pham, Hanif Esmail, Abdel Babiker, James R. Carpenter

**Affiliations:** 1 MRC Clinical Trials Unit at UCL, London, UK; 2 Imperial Clinical Trials Unit, Imperial College London, London, UK; 3 HRB Clinical Research Facility Cork, Cork, Ireland; 4 School of Public Health, University College Cork, Cork, Ireland; 5 Institute for Global Health, University College London, London, UK

**Keywords:** COVID-19, Estimand, Randomised trial, Intercurrent events, Truncation-by-death

## Abstract

When designing a clinical trial, explicitly defining the treatment *estimands* of interest (*that* *which is to be estimated*) can help to clarify trial objectives and ensure the questions being addressed by the trial are clinically meaningful. There are several challenges when defining estimands. Here, we discuss a number of these in the context of trials of treatments for patients hospitalised with COVID-19 and make suggestions for how estimands should be defined for key outcomes. We suggest that treatment effects should usually be measured as differences in proportions (or risk or odds ratios) for outcomes such as death and requirement for ventilation, and differences in means for outcomes such as the number of days ventilated. We further recommend that truncation due to death should be handled differently depending on whether a patient- or resource-focused perspective is taken; for the former, a composite approach should be used, while for the latter, a while-alive approach is preferred. Finally, we suggest that discontinuation of randomised treatment should be handled from a treatment policy perspective, where non-adherence is ignored in the analysis (i.e. intention to treat).

## Background

As of 8 July 2020, over 1600 clinical trials have been registered to evaluate different treatment options for coronavirus disease (COVID-19) [1, 2]. Evidence appraisal and synthesis to identify which treatments are best will require that trials address meaningful questions (for instance, by measuring clinically relevant outcomes) and that results can be meaningfully compared across trials (for instance, by standardisation of outcomes across trials). Core outcome sets have identified all-cause mortality and respiratory support as the key outcomes to be measured in trials of in-hospital treatments for COVID-19 [3, 4]. Hospital resource outcomes, such as length of stay, time in intensive care units (ICUs), and time on ventilators, have also been recommended [5–7].

However, to ensure that trials address meaningful questions, and to facilitate comparisons across trials, it is also necessary to define the *estimand* of interest. An estimand is a precise definition of the treatment effect to be estimated [8]; careful consideration of the estimand can help to ensure that research objectives are clearly stated, address a clinically meaningful question, and are aligned with the study procedures, including trial design, data to be collected, and planned statistical analysis.

## Main text

There are several challenges around defining estimands for trials in patients hospitalised for COVID-19, and inappropriate choices for these estimands may lead to results that are difficult to interpret or misleading [8–23]. For example, consider the number of days on a ventilator. This outcome is important both as a marker of patient health and to healthcare systems as a whole, as any reduction means more ventilators are available for other patients. A key challenge when defining an estimand for this outcome is that patients who die early may have fewer days on a ventilator, which can make interventions with higher mortality rates appear more effective. Clearly, a shorter time on a ventilator due to death is not a good outcome, and this fact should be reflected in the choice of estimand. For example, a composite outcome could be used, where patients who die are assigned a poor value. However, this approach may not be helpful for evaluating whether an intervention can help healthcare systems by freeing up ventilators to be used for other patients, as it does not give the actual number of ventilator days saved. As such, an alternative strategy would be required to understand to what extent the intervention could free up ventilators for use in other patients.

Different estimands can produce different conclusions around treatment benefits and harms [13, 15], and estimands that are not clearly defined (for instance, that do not specify how data from patients who die will be handled) can lead to confusion around results [8]. In light of the core outcomes identified for in-hospital treatments for COVID-19, we consider three important aspects of how estimands for these outcomes should be defined: (i) choice of treatment effect measure to compare outcomes between treatment groups, (ii) how to deal with truncation of outcome data due to death, and (iii) how to deal with treatment discontinuation. Our suggested strategies are provided in Table 1; the rest of this article explains the rationale for these choices, with the aim of helping trialists to incorporate appropriate estimands into their own trials of COVID-19.

**Table 1:** Suggested strategies for defining estimands for core COVID-19 outcomes^a^

Objective (outcome in bold). Objectives relate to the effect of treatment if introduced into a healthcare system	Treatment effect	Truncation by death	Treatment discontinuation
Evaluate the effect of treatment on **mortality**^†^	Difference in proportion dying by a specific time point (or risk ratio or odds ratio)	NA	Treatment policy strategy^b^
Evaluate the effect of treatment on the **requirement for ventilation/oxygen/ICU** as a measure of patient benefit	Difference in proportion affected by a specific time point (or risk ratio or odds ratio)	Composite strategy: death is set as failure	Treatment policy strategy^b^
Evaluate the effect of treatment on the **requirement for ventilation/oxygen/ICU** from a healthcare systems perspective	Difference in proportion affected by a specific time point (or risk ratio or odds ratio)	While-alive strategy: data from when the patient is alive is used (e.g. did they require ventilation prior to death?)	Treatment policy strategy^b^
Evaluate the effect of treatment on the **number of days in hospital/on a ventilator/on oxygen/in ICU** as a measure of patient benefit	Difference in means or restricted mean time	Composite strategy: outcome is defined as the number of days alive and out of hospital/off a ventilator/off oxygen/out of ICU within a given time period	Treatment policy strategy^b^
Evaluate the effect of treatment on the **number of days in hospital/on a ventilator/on oxygen/in ICU** from a healthcare systems perspective	Difference in means or restricted mean time	While-alive strategy: data from when the patient is alive is used (e.g. patients are counted as not on a ventilator from point of death)	Treatment policy strategy^b^

^a^Other estimand aspects (treatment, population, other intercurrent events) also need to be specified in order to have fully defined estimands

^b^Can be implemented using intention-to-treat analysis, where all randomised patients are included, and analysed according to their randomised group

^†^Effect to individual patients or to healthcare systems as a whole on mortality

## Description of estimands

The recent ICH-E9 addendum, which sought to clarify the role of estimands in clinical trials, defines an estimand as “a precise description of the treatment effect reflecting the clinical question posed by a given clinical trial objective. It summarises at a population level what the outcomes would be in the same patients under different treatment conditions being compared” [8]. Since different estimands may be of interest to different stakeholders (e.g. patients, clinicians, healthcare managers, regulatory agencies), it is vital to properly define them when designing a trial, so that the subsequent data collection and planned statistical analyses can be aligned with the key questions being asked by the trial.

An estimand consists of the following five components: (1) the treatment regimens to be compared, (2) the patient population of interest, (3) the outcome definition, (4) a population-level summary denoting how outcomes between treatment arms will be compared (i.e. the type of treatment effect, such as a hazard ratio, difference in percentage points, difference in means), and (5) how post-randomisation (intercurrent) events which may influence interpretation of the treatment effect, such as mortality or treatment discontinuation, will be handled.

Following the evaluation of a number of published COVID-19 trials [24], we focus here on the estimand components which require the most attention to ensure appropriate interpretation of COVID-trials: (i) how outcomes should be compared between treatment groups (population-level summary measure), (ii) how to handle truncation by death (intercurrent event), and (iii) how to handle treatment discontinuation (intercurrent event). We focus on the outcomes defined in the ‘meta’ core outcome set (mortality, respiratory support) [3], as well as key hospital resource outcomes such as time in hospital, time in ICU, and time on a ventilator.

We note that, in addition to the aspects described below (population-level summary, truncation by death, treatment discontinuation), investigators would also need to define the other aspects listed above to form a complete estimand (i.e. the treatment regimes, patient population, and handling of other intercurrent events, such as the use of non-trial treatments). For instance, certain interventions may be targeted at specific patients, such as those already on respiratory support, or those with certain co-morbidities, such as diabetes. A precise definition of these aspects is essential for appropriate interpretation and comparison of results.

## Trial objective

In this paper, we focus on the objective of evaluating the effect of treatment if it was introduced into a healthcare system, i.e. to address the question “if this intervention were introduced as the standard of care into routine clinical practice, how much benefit would there be to patients?”

For outcomes involving health resource utilisation (e.g. requirement for ventilation/oxygen/ICU or the number of days in hospital/on a ventilator/on oxygen/in ICU), we consider this benefit from two different perspectives: (i) the benefit to individual patients and (ii) benefits to healthcare systems as a whole, through reductions in resource use which can then be used for other patients. Both perspectives are important to understanding the benefit of introducing the intervention into a healthcare system, and so multiple estimands should be used. We discuss this further below.

Although we feel the objective outlined above is the most important in identifying treatments which can help to protect against impacts of the pandemic, we note that other objectives may also be of interest in certain settings; in this case, alternative estimands could be defined.

## Population-level summary (treatment effect) used to compare outcomes between treatment groups

Table 2 provides a summary of the common treatment effect measures that could be used to analyse the outcomes considered here. These include the following: (a) hazard ratio, (b) difference in proportions (or risk or odds ratio) at a specific time point (e.g. percentage surviving to hospital discharge or percentage still alive at day 28), (c) difference in means or difference in restricted mean time (e.g. difference in the mean number of days in hospital up to day 30), and (d) difference in medians.

**Table 2:** Summary of treatment effect measures

Treatment effect measure	Explanation
Hazard ratio	The hazard ratio provides a weighted average of the hazards across all follow-up time points. In some cases, this interpretation can be difficult to understand; in Fig. 1, the hazard ratio is 0.90, indicating some treatment benefit. However, there is no difference in events between treatment groups at 28 days, and the hazard ratio gives no indication of how much additional time is conferred through the intervention.
Risk difference at a specific time point	A difference in percentage points (or risk or odds ratio) at a specific time point provides an overall measure of benefit within that time period. However, it does not take into account the timing of events within that time span, and so, its appropriateness will depend on whether trial objectives relate to the occurrence of an event within a time period, or altering the time until an event.
Difference in means or difference in restricted mean time	A difference in means provides a measure of benefit across the entire distribution, while the difference in restricted mean time (commonly referred to as ‘restricted mean survival time’) provides a measure of benefit within a certain time period; for instance, in Fig. 1, the difference in restricted mean time is − 1.0 days, meaning that over a 28-day period, patients in the intervention group had on average 1 additional day before an event. The two measures will typically differ, with earlier restrictions typically leading to greater differences. When feasible, for outcomes such as the number of days in hospital/on a ventilator/on oxygen/in ICU, using the mean or a later restriction is usually more informative.
Difference in medians	A difference in medians provides a measure of benefit seen at the midpoint of the distribution. Although this can be informative in some settings, it can also mask what happens in other parts of the distribution.

**Fig. 1:**
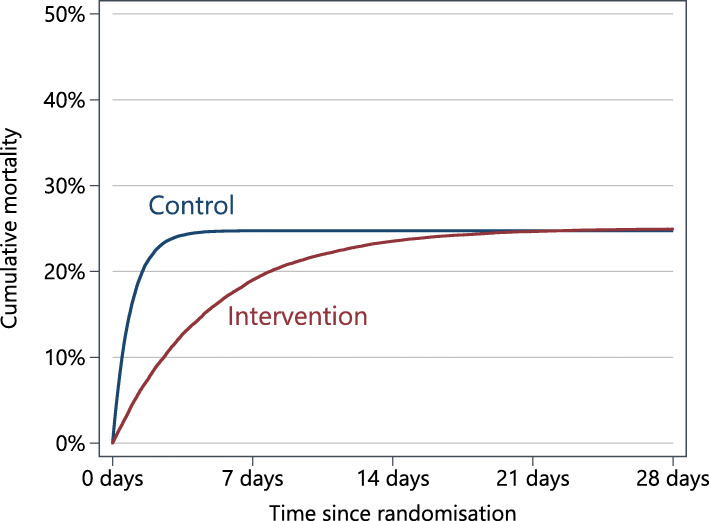
Mortality in a fictional trial. Hazard ratio = 0.90; difference in percentage points at day 28 = 0.0; difference in restricted mean survival time up to day 28 = 1.0 days

An explanation of each measure is provided in Table 2. We highlight the type of information conveyed through each treatment effect, using Fig. 1 as an example. In this fictitious trial, 25% of patients in both treatment groups experience the event of interest by day 28; however, patients in the intervention group take longer to experience the outcome than those in the control group.

The most appropriate measure of treatment benefit for each outcome will depend on the nature of the outcome and the trial objective, for instance, whether the aim of treatment is to prevent the outcome from occurring entirely within the follow-up time frame or whether the outcome will occur for most patients and the goal is to increase (or reduce) the time until the event occurs.

For instance, consider in-hospital mortality, recommended by the meta core outcome set [3], or mortality up to 28 days as used in other trials [25]. Here, the goal is to prevent the occurrence of death entirely within this time period. Consider the fictitious example in Fig. 1, there is no mortality benefit at 28 days, but the treatment effects from a hazard ratio (0.90) and the difference in restricted mean time (− 1.0 days) suggest some measure of benefit, as treatment extends survival by a modest amount. However, the aim here is not to extend survival by 1 day, or even 10 days; it is to prevent mortality entirely. Therefore, while treatment effects such as hazard ratios or restricted mean time may be appealing, they are not in line with the objectives of COVID-19 trials; a difference in percentage points (or a risk or odds ratio) should be used instead for short-term mortality endpoints. The same objective applies to the requirement for ventilation/oxygen/ICU outcomes, and so a difference in percentage points should be used for these as well.

Conversely, for an outcome such as time to hospital discharge, even a small difference in timing could be clinically important. For instance, a mean reduction of 0.5 days would mean that for every 100 patients treated, 50 extra bed days become available. This would be beneficial for healthcare system resources. In contrast, a hazard ratio, difference in percentage points, or difference in medians may be less meaningful, as they do not provide direct information about the actual number of hospital bed days saved. A hazard ratio or a difference in percentage points only provides information about the probability of being discharged, rather than the time to discharge. Likewise, a difference in medians may not provide an accurate picture of the overall amount of hospital resource saved, as it describes the effect in the middle of the distribution (at the 50th percentile), and it may miss treatment effects seen in other parts of the distribution.

## Approaches to handling outcomes truncated by death (intercurrent event)

Most trials of in-hospital treatments for COVID-19 will have non-negligible mortality rates. For example, in the trial by Cao et al. [25], 19.2% of patients died by day 28 in the intervention group, compared with 25.0% in the control group. This can pose a challenge to the interpretation of certain outcomes. For instance, consider the outcome ‘days until hospital discharge’: for patients who die while in hospital (and thus are not discharged), the relevant outcome data no longer exists after death, and so, it is unclear how the outcome should be defined. We refer to this as data truncated by death.

It can be argued that when the intervention reduces mortality, other outcomes become less important, and hence, the approach used to handle truncation by death in these outcomes matters less. However, even if a treatment reduces mortality, outcomes such as intensive care unit (ICU) stay or days on a ventilator can help to clarify planning of healthcare resources. Further, it is not always clear whether an intervention does improve survival; in the trial by Cao et al. [25], the confidence intervals are consistent with both no effect and a large effect, and so, other outcomes can be important to help weigh the benefits of treatment.

It is therefore important to ensure truncation by death is handled appropriately in the estimand, as inappropriate approaches can be misleading, for instance, by making treatments with higher mortality rates appear to be more effective (e.g. patients may have fewer days in hospital simply due to a shorter survival time). In particular, we advise against an analysis amongst survivors (where patients who survived in the control group are compared against patients who survived in the intervention group). When treatment affects mortality (as is typically the aim in COVID-19 trials), this approach can introduce bias due to the differences between patients who survive in each group [26].

The main approaches that have been proposed to deal with truncation by death are summarised in Table 3. We discuss our suggested approaches below and in Table 1, separately for the two perspectives related to outcomes involving health resource utilisation (e.g. requirement for ventilation/oxygen/ICU or the number of days in hospital/on a ventilator/on oxygen/in ICU): (i) the benefit to individual patients and (ii) benefits to healthcare systems as a whole, through reductions in resource use which can then be used for other patients. We note that both perspectives can be important, and so, multiple estimands could be used.

**Table 3:** Summary of strategies for handling intercurrent events in trials for COVID-19. Suggestions relate to an objective of evaluating the effect of treatment if they were introduced into a healthcare system

Strategy	Explanation	Truncation-by-death^**a**^	Treatment discontinuation^**b**^
Treatment policy	Measures the effect of the original decision to undertake a treatment, where the intercurrent event (e.g. discontinuation) is taken to be part of the treatment strategy. Cannot be used for terminal events, such as mortality.	Not applicable; relevant outcome data does not exist.	Recommended strategy, as it most closely links to the objective of evaluating the effect of treatment if introduced into a healthcare system.
Composite	The outcome definition is modified to incorporate the intercurrent event, e.g. ‘requirement for ventilation’ is modified to ‘requirement for ventilation or death’.	Recommended strategy for *patient benefit perspective*, as it ensures death equates to a poor outcome. Care is required to ensure the outcome remains interpretable/clinically meaningful.	Not recommended, as the outcome becomes less interpretable/clinically meaningful.
Hypothetical	Measures the effect of treatment in a hypothetical setting where the intercurrent event would not occur, e.g. the treatment effect if there was no discontinuation.	Not recommended; applies to a hypothetical setting which will never exist (no patients die), and so is difficult to interpret.	Recommended in a secondary estimand for discontinuation due to external factors (e.g. supply issues/lack of PPE), to evaluate the effect of treatment in settings where there was no supply issues/lack of PPE.
While alive/while on treatment	Uses data prior to the occurrence of the intercurrent event; e.g. for ICU days, the number of days a patient was in ICU before they died would be used.	Recommended strategy for *healthcare systems perspective*, as it provides the real-world resource savings due to treatment.	Not recommended, as estimand becomes less interpretable/clinically meaningful.
Principal stratum	Measures the effect of treatment in the (unknown) subpopulation of patients for whom the intercurrent event would not occur.	Not recommended, as interest for COVID-19 trials is likely to be a treatment effect in the entire population of patients, rather than in an unknown subpopulation.	Not recommended, as interest for COVID-19 trials is likely to be a treatment effect in the entire population of patients, rather than in an unknown subpopulation.

^a^Truncation-by-death acts as an intercurrent event for all outcomes considered in this manuscript except for all-cause mortality

^b^Treatment discontinuation acts as an intercurrent event for all outcomes considered in this manuscript

### Patient benefit perspective

When the objective is to identify patient benefit, it is important to use an approach that does not allow the occurrence of death to result in a ‘good’ outcome (e.g. not to count patients who die before requiring ventilation as a success for the outcome ‘requirement for a ventilator’).

For outcomes such as the requirement for ventilation, a simple approach is to use a composite strategy, where patients who die are set as a failure; that is, the outcome is redefined to be ‘requirement for a ventilator or death’. This approach ensures death is appropriately reflected in the outcome measure as a poor result; however, it does change the interpretation of the estimand, and any observed treatment benefit could be due to either reduction in ventilation use or death.

For outcomes such as the number of days in hospital or on a ventilator, a composite strategy will ensure that death equates to a bad outcome; however, the issues around the change in interpretation become more pronounced. If patients who die are assigned a value of 28 days in hospital, then it is difficult to determine exactly what a difference of − 2 days between treatment groups means, as this does not correspond to the actual difference in hospital days. An alternative approach is to redefine the outcome from a negative outcome (days in hospital) to a positive outcome (days out of hospital), which makes it easier to incorporate death using a composite strategy, i.e. the outcome could be redefined as the number of days alive and out of hospital within a certain time period (or off oxygen/off a ventilator/out of ICU); for example, a patient who goes into ICU on day 3 and then dies on day 8 would be defined as having 3 days alive and out of ICU (instead of 5 days in ICU).

### Healthcare systems perspective

When the objective is to identify the benefit to healthcare systems (through resource savings that could allow additional patients to be treated), a composite strategy is not relevant, as it does not correspond to the actual amount of resources saved, i.e. it does not help to inform the actual number of additional bed or ventilator days that would become available in real life. As such, we recommend the while-alive strategy to address objectives related to the healthcare systems perspective, as this approach provides the real-world resource savings due to treatment, which are required to inform planning or modelling of healthcare capacity. However, because this approach can be affected by differences in survival between treatment groups, it should be interpreted in light of the overall mortality results.

## Approaches to handling discontinuation of randomised treatment (intercurrent event)

Some patients may discontinue their randomised treatment early or may not receive treatment at all. For example, in the trial by Cao et al., a small subset of patients in the intervention group did not receive any dose of study drug [25]. Strategies for handling treatment discontinuation are summarised in Table 3.

We differentiate between treatment discontinuation due to internal factors [22], such as adverse events, problems with the route of administration, or a clinician’s decision to discontinue for end-of-life care, and discontinuation due to external factors [22], for example, because drug supply issues mean the treatment is no longer available or a lack of personal protective equipment means that clinicians cannot administer the treatment as intended.

Our suggested strategy is summarised in Table 1. For treatment discontinuation due to internal factors, we recommend a treatment policy strategy (corresponding to an intention-to-treat analysis), as these events are likely to occur in practice; hence, this strategy most closely links to the objective of evaluating the effect of treatment if introduced into a healthcare system.

However, for treatment discontinuation due to external factors (e.g. drug supply issues, lack of personal protective equipment), it is easy to imagine settings where this would not occur in practice (once the drug supply issues were resolved, or in countries with adequate personal protective equipment). Therefore, we recommend the main estimand be defined using a treatment policy strategy, but that a secondary estimand using a hypothetical strategy could be considered, which addresses the effect of treatment had there been adequate supply of drugs or personal protective equipment. In some instances, however, the hypothetical strategy may be more appropriate for the primary estimand (for instance, if these external factors are very unlikely to recur).

## Discussion

There is growing recognition that focusing on estimands at the design stage can help to clarify trial objectives and ensure that trial methods align with these objectives. We have proposed a set of estimands that could be used for trials of in-hospital treatments for COVID-19 (Table 1). We recommend that a difference in percentage points (or risk or odds ratios) be used for outcomes such as mortality and requirement for ventilation, and a difference in means (or restricted mean time) be used for outcomes such as the number of days in hospital/on a ventilator. We suggest that truncation due to death should be handled differently depending on whether a patient perspective or a healthcare systems perspective is taken; for a patient perspective, a composite approach should be used, while for a healthcare systems perspective, a while-alive strategy should be taken. Finally, we suggest that treatment discontinuation should be handled using a treatment policy strategy, i.e. ignoring the non-adherence in the analysis.

The statistical methods and data collection should be aligned to the chosen estimands to ensure that key questions can be answered. The estimands proposed in this article can be addressed using simple statistical methods: for the treatment policy strategy for treatment discontinuation or non-adherence, an intention-to-treat analysis can be used, where all patients are included in the analysis and analysed according to their allocated treatment group [27, 28]; for a composite strategy for truncation due to death, outcomes can easily be redefined as composites, and standard statistical methods employed; and a while-alive strategy for a healthcare systems perspective can be easily implemented using standard statistical approaches, though care should be taken, as some common statistical methods (such as repeated-measures mixed-effects models) can inadvertently impute data post-death [29]. Finally, the estimands proposed here require minimal additional data collection. Outcome data should continue to be collected even after treatment discontinuation, as required for the treatment policy estimand [8], and certain outcomes may require additional data collection even after hospital discharge. For example, the outcome ‘days alive and out of hospital’ would require an assessment of whether the patient may have been re-admitted after initial discharge, and the outcome ‘mortality within 28 days’ would require an assessment of the patient’s mortality status after hospital discharge for patients who were discharged before day 28.

We note that not all the outcomes or estimands discussed here will be appropriate in all trials. For instance, in trials enrolling only severely ill patients on a ventilator, the outcome ‘requirement for ventilation’ is clearly not useful. However, the issues discussed in this article are likely to be universal across most trials of treatments for patients hospitalised for COVID-19, and so consideration of these issues will enable investigators to identify the most appropriate estimands for their own trial.

There are a number of approaches to handling the issues outlined in this paper that we have not considered [30, 31]. The methods outlined in this paper were not intended to be comprehensive, and alternative approaches may be more appropriate for some trials. The important thing is for trialists to identify strategies for handling the issues outlined in this paper, as well as any issues unique to their trial, that lead to a clear estimand which is clinically meaningful and addresses the trial’s objective.

We have limited this paper to outcomes identified within a meta core outcome set [3] and key hospital resource outcomes. Other outcomes are also commonly used, and the same considerations outlined here could be applied to define estimands for these outcomes as well. For instance, some trials may conduct long-term follow-up of patients (as short-term outcomes may not always extrapolate to long-term follow-up), and therefore include a long-term mortality outcome, such as ‘mortality at two years’; here, the specific timing of the event may be more relevant than it is over a short-term follow-up (e.g. an increase in survival of 2 months over a 2-year period may be clinically meaningful, whereas an increase of 2 days over a 30-day period is likely not), and so a treatment effect measure such as a difference in restricted mean time may be a useful approach.

One outcome in common use is a 7-category ordinal scale at a fixed time point (e.g. 14 days after randomisation), which includes categories based on mortality, respiratory support, and hospital discharge status [6, 32–34]. Although this outcome contains more information regarding patient status at a given time point than each outcome considered alone, it can be difficult to interpret. For example, it is impossible to determine whether a beneficial treatment effect is because the intervention increases the number of patients who survive to hospital discharge instead of dying, or because it increases the number of patients who require non-invasive instead of invasive mechanical ventilation. In our view, it is preferable to define separate estimands for each outcome component individually (i.e. separate estimands for mortality, time in hospital, requirement for ventilation), to ensure results are both interpretable and clinically meaningful [35].

Investigators may sometimes choose their estimand based on statistical properties, rather than clinical relevance. For instance, sometimes the median is recommended over the mean due to perceived skewness or a lack of normality of the data, or the hazard ratio is recommended over a difference in percentage points because it is seen to be more statistically efficient. However, choosing an estimand based on statistical considerations can be problematic if the chosen estimand is not clinically meaningful [9]. The hazard ratio may have higher statistical power than a difference in percentage points for mortality at 28 days in some settings; however, this is not necessarily an advantage if the estimated treatment effect is not clinically relevant (as is the case in Fig. 1). Similarly, the median considers the treatment effect at only a single point in the distribution and may hide important effects seen elsewhere. Although statistical efficiency is important, it should not come at the cost of a clinically relevant estimand.

Many trials of in-hospital treatments for COVID-19 have already started. Due to the extremely tight timelines and difficult circumstances involved, these trials may not have defined their target estimand or specified how they plan to handle issues such as truncation by death. In these trials, it is still important to define estimands, even if done while the study is ongoing, as this will help to clarify study objectives and ensure the statistical analysis approach is in line with those objectives. Any changes to outcomes (e.g. change the outcome ‘requirement for ventilation’ to ‘requirement for ventilation, or death’) or to methods should be updated in the trial registry, protocol, and statistical analysis plan as appropriate. Any changes or new outcomes should be clearly labelled as post hoc in the trial publication, with a rationale for the change provided [28, 36].

## Conclusion

Specifying the estimand in COVID-19 trials can help to ensure that trials are addressing the right question and that results are clinically meaningful.

## Data Availability

Not applicable
